# Dinuclear thiazolylidene copper complex as highly active catalyst for azid–alkyne cycloadditions

**DOI:** 10.3762/bjoc.12.151

**Published:** 2016-07-21

**Authors:** Anne L Schöffler, Ata Makarem, Frank Rominger, Bernd F Straub

**Affiliations:** 1 Organisch-Chemisches Institut, Universität Heidelberg, Im Neuenheimer Feld 270, D-69120 Heidelberg, Germany

**Keywords:** catalysis, click, copper, CuAAC, *N*-heterocyclic carbene, thiazole

## Abstract

A dinuclear *N*-heterocyclic carbene (NHC) copper complex efficiently catalyzes azide–alkyne cycloaddition (CuAAC) “click” reactions. The ancillary ligand comprises two 4,5-dimethyl-1,3-thiazol-2-ylidene units and an ethylene linker. The three-step preparation of the complex from commercially available starting compounds is more straightforward and cost-efficient than that of the previously described 1,2,4-triazol-5-ylidene derivatives. Kinetic experiments revealed its high catalytic CuAAC activity in organic solvents at room temperature. The activity increases upon addition of acetic acid, particularly for more acidic alkyne substrates. The modular catalyst design renders possible the exchange of *N*-heterocyclic carbene, linker, sacrificial ligand, and counter ion.

## Introduction

**Scheme 1:**
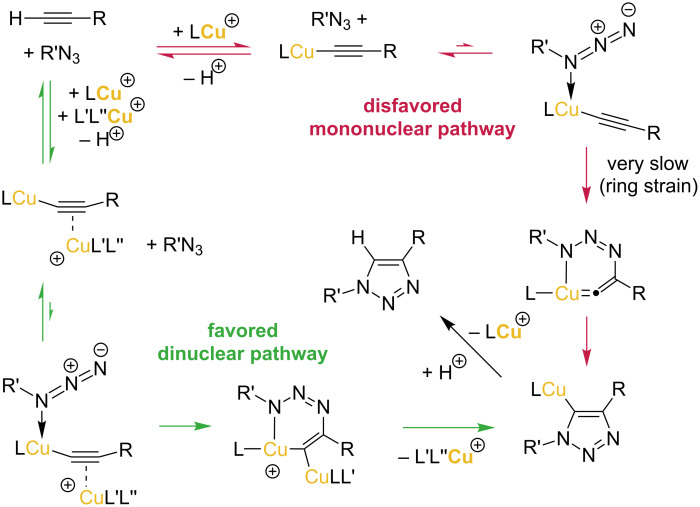
Disfavored mononuclear pathway and favored dinuclear pathway in the CuAAC click reaction, according to the mechanistic proposal of reference [37]. R, R’ = alkyl, aryl, silyl, carbonyl groups; L = NHC; L’ = NHC or solvent; L’’ = solvent, acetylide, carboxylate, halide.

The copper-catalyzed azide–alkyne cycloaddition (CuAAC) is one of the most important “click” reactions for the facile covalent linking of two molecules [1–3]. In 2002, the research groups of Meldal and Sharpless independently discovered the strongly rate-enhancing effect of copper(I) salts for azide–alkyne cycloadditions. The 1,4-disubstituted 1,2,3-triazoles are formed exclusively with essentially quantitative conversion and under mild reaction conditions [4–6]. The CuAAC reaction has found broad application in the preparation of peptide-conjugates [7–8], multicomponent syntheses [9], preparation of hydrogels, microgels and nanogels [10], (anion) supramolecular chemistry [11–12], in medicinal chemistry [13], therapeutics, biomaterials and bioactive surfaces [8,14], imaging of biochemical processes [15], localization of bioactive compounds inside living cells [16], syntheses of small-molecule screening libraries [17], catenane and rotaxane syntheses [18], in reactions under continuous flow processing [19], polymer and surface science [20–26], nucleoside, nucleotide, and oligonucleotide chemistry [27–29], in fluorogenic reactions [30], and in the syntheses of dendrimers [25,31]. The detailed mechanism of this reaction and the exact role of copper(I) and the species involved in the catalytic cycle had remained unclear for many years. The first proposals for the mechanism suggested the participation of only one copper(I) atom in the key elementary steps. In 2005, Fokin and Finn determined the reaction rate of a CuAAC reaction to be second order in the concentration of copper [32]. Since then, the understanding of the mandatory role of dinuclear copper complexes as catalyst intermediates has vastly improved (Scheme 1) [33–37]. In 2013, the Fokin group provided evidence for the dicopper pathway of CuAAC reactions by a series of kinetic and isotopic labeling studies [34]. In 2015, the Bertrand group prepared, isolated, and structurally characterized molecular dicopper acetylide complexes, and investigated their reactivity towards azide substrates [36].

For standard CuAAC reactions, copper(I) carboxylates [5,38], mononuclear copper(I) phosphine carboxylate complexes [39] or copper(I) salts are an ideal compromise of low catalyst cost and high catalyst activity. However, CuAAC reactions of some substrates are not compatible with heterogeneous catalysis at the surface of insoluble copper(I) compounds. Instead, they depend on highly active molecular catalysts under homogeneous reaction conditions. Our research group has already established molecularly defined dicopper catalysts with unprecedented activity under diluted conditions with low catalyst loading [37,40]. Thus, we aimed at an even more facile synthesis of dicopper complexes with bis-*N*-heterocyclic carbene ancillary ligands.

## Results and Discussion

**Figure 1:**
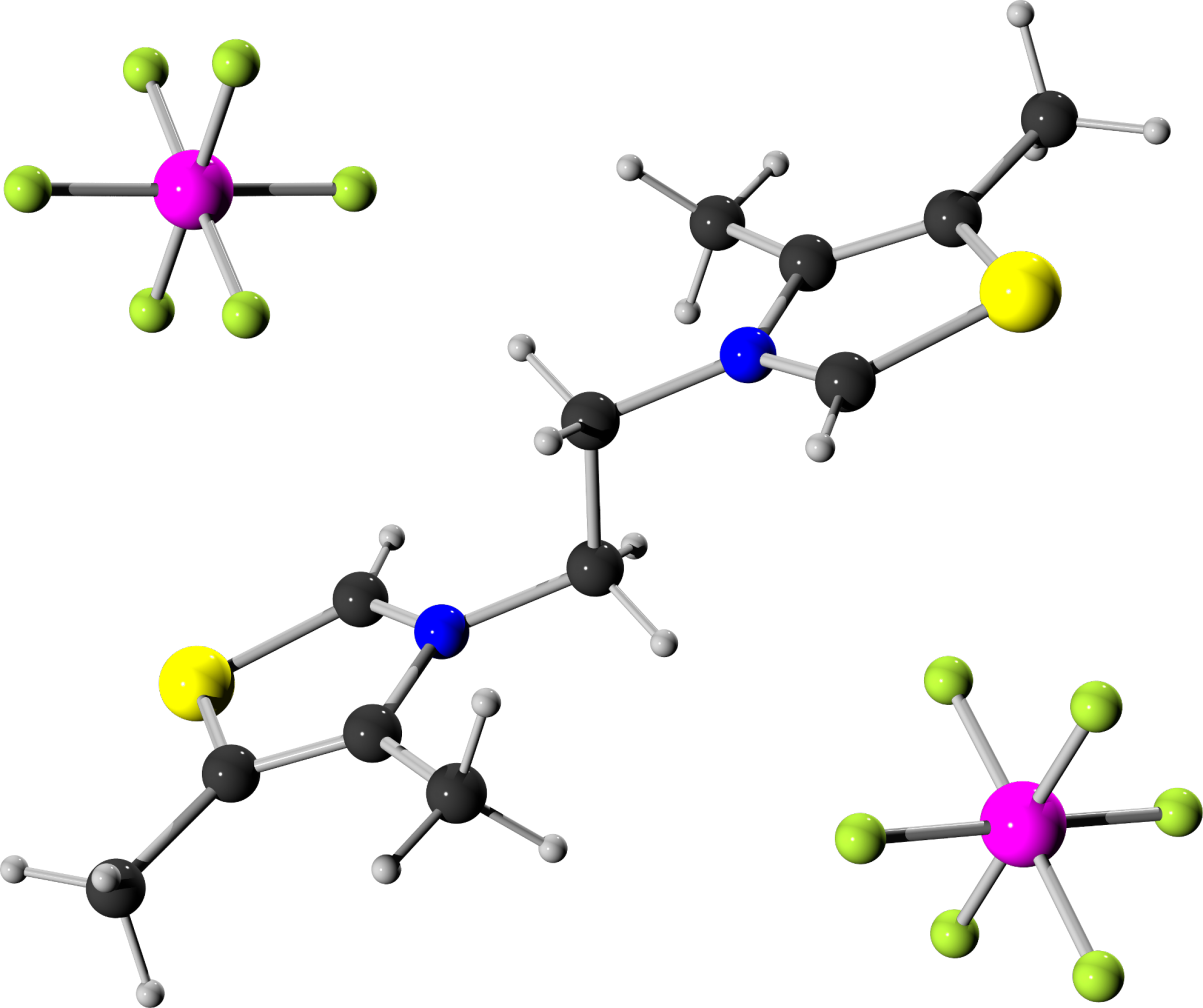
Ball-and-stick model [42–43] of a single crystal X-ray structure of hexafluorophosphate salt **1b** (CCDC 1472789). Color code: black carbon, grey hydrogen, yellow sulfur, blue nitrogen, magenta phosphorus, green fluorine.

We herein report the synthesis of an ethylene-linked bisthiazol-2-ylidene dicopper(I) complex **2** that features high catalytic activity in CuAAC reactions. The advantage of this new complex **2** in comparison to dicopper complexes previously described by our research group [40] is its less time-consuming and more cost-efficient synthesis. Commercially available, inexpensive 4,5-dimethylthiazole is used as azole starting material instead of 4-aryl-1,2,4-triazoles. The precursor **1a** for the NHC ancillary ligand is synthesized via a double S_N_2-reaction of two equivalents of thiazole derivative with 1,2-dibromoethane. In order to avoid the presence of halide ions as inhibitory ligands for copper(I) [2,41], bisthiazolium hexafluorophosphate **1b** was obtained by a salt metathesis from bromide salt **1a** with aqueous hexafluorophosphoric acid (Figure 1).

**Scheme 2:**
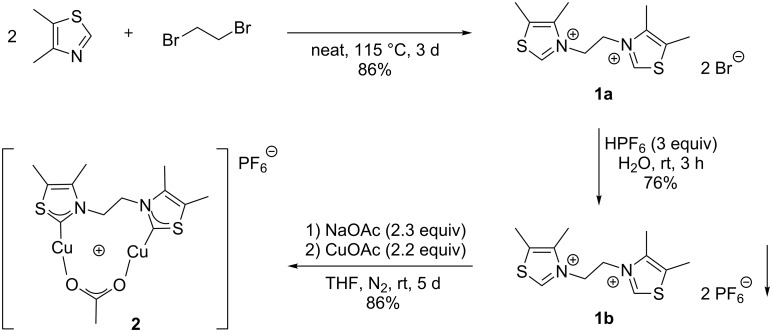
Synthesis of dinuclear copper complex **2**.

The final step is the reaction with copper(I) acetate and sodium acetate as additional base in order to deprotonate the thiazolium salt **1b** and to form the bisthiazolylidene copper(I) complex **2**. Due to the relatively high acidity of the thiazolium precursor (p*K*_a_ ≈ 18 [44]), a weak base such as sodium acetate yields small equilibrium concentrations of thiazol-2-ylidene. The latter then irreversibly binds to copper(I) ions (Scheme 2). The structure of the resulting complex **2** is presumably similar to the previously reported dinuclear bis(1,2,4-triazol-5-ylidene)copper(I) complexes that had been synthesized, characterized, and structurally characterized in our group [40]. The similarity of NMR-spectroscopic data of the 1,2,4-triazol-5-ylidene and the 1,3-thiazol-2-ylidene dicopper complex indicate that the complexes of both NHC ligand types consist of a bis-NHC ligand, two copper(I) ions and a labile acetate ligand that bridges the metal centers. The thiazolylidene complex **2** is air-stable in the solid state for at least several days. Stability tests in solution were taken under an atmosphere of nitrogen. Small amounts of a brown precipitate were formed in solution after one day. However, the NMR spectra showed no changes even after one week and in the presence of acetic acid. Therefore, we assume that complex **2** is quite robust against oxidization.

**Figure 2:**
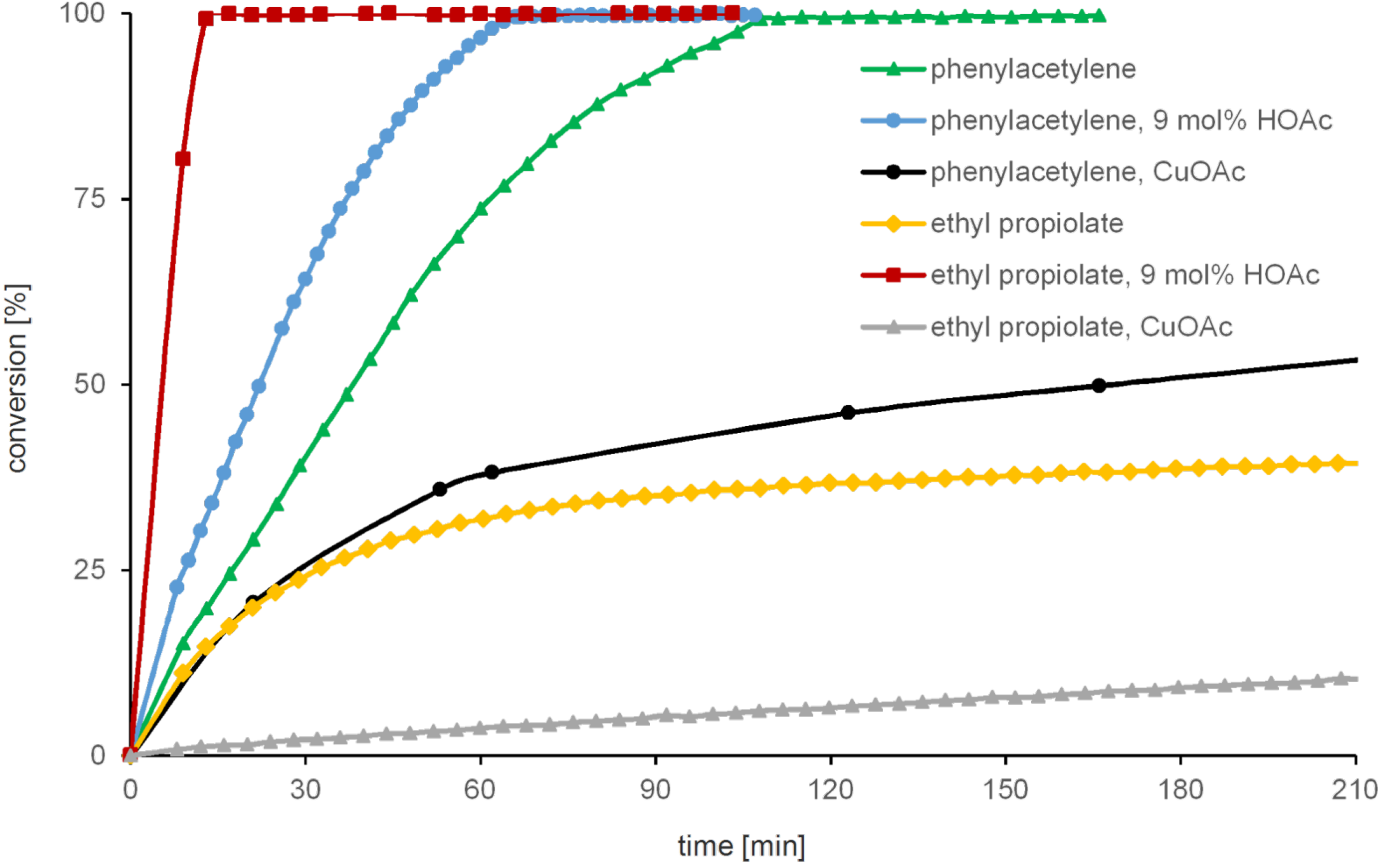
Time-conversion-diagram of the CuAAC reaction of benzyl azide with either phenylacetylene or ethyl propiolate in the presence of copper complex **2** (1.8 mol % for reaction with phenylacetylene, 0.9 mol % for reaction with ethyl propiolate) or in presence of CuOAc (saturated homogeneous solution) under an atmosphere of nitrogen in CD_2_Cl_2_ at rt (conversion referred to benzyl azide); reaction with phenylacetylene: green triangles (complex **2** without additional HOAc), blue dots (complex **2** in presence of 9 mol % additional HOAc) and black dots (CuOAc solution); reaction with ethyl propiolate: yellow diamonds (complex **2** without additional HOAc), red squares (complex **2** in presence of 9 mol % additional HOAc) and grey triangles (CuOAc solution).

To test the catalytic performance of complex **2** with the help of continuous NMR spectroscopy, the reaction of benzyl azide with either phenylacetylene or ethyl propiolate in deuterated dichloromethane at room temperature was used (Table 1 and Figure 2). Due to the highly exothermic nature of the triazole formation, a high dilution of the reaction mixture and low catalyst loadings are necessary to prevent a thermal runaway. In order to compare the catalytic activity of complex **2** with conventional catalysts a kinetic study with copper(I) acetate was performed. All kinetic experiments were carried out under an atmosphere of nitrogen because of the air-sensitivity of complex **2** in solution (see Supporting Information File 1 for the detailed procedure).

**Table 1:** CuAAC reaction of benzyl azide and terminal alkynes with complex **2** or CuOAc as catalyst in absence or presence of added acetic acid.

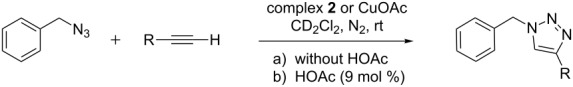				

Entry	Alkyne	Catalyst	Additional HOAc	Half conversion time

1	phenylacetylene	1.8 mol % complex **2**	–	37 min
2	phenylacetylene	1.8 mol % complex **2**	9 mol %	22 min
3	phenylacetylene	CuOAc (homogeneous solution)	–	166 min
4	ethyl propiolate	0.9 mol % complex **2**	–	≈ 15 h^*^
5	ethyl propiolate	0.9 mol % complex **2**	9 mol %	6 min
6	ethyl propiolate	CuOAc (homogeneous solution)	–	≈ 19 h^*^

*extrapolated half conversion time.

The reaction with phenylacetylene and 1.8 mol % copper complex **2** (Table 1, entries 1 and 2) reaches 50% conversion within 37 min (without acetic acid, green triangles in Figure 2) and is slightly accelerated by addition of acetic acid (half conversion within 22 min, blue dots). In contrast, the half conversion time for this reaction catalyzed by a saturated homogeneous solution of copper(I) acetate in deuterated dichloromethane is about 3 h (Table 1, entry 3, black dots in Figure 2). Therefore, the reaction with complex **2** is about 4.5 times (without HOAc) to 7.5 times (with HOAc) more effective compared to the homogeneous reaction with copper acetate. Under heterogeneous catalytic conditions, however, larger amounts of commercially available CuOAc powder with vivid stirring or shaking of the reaction mixture give rise to rapid CuAAC conversion. Thus, the molecular NHC dicopper catalyst complexes excel in homogeneous CuAAC reactions [40], while CuOAc excels in heterogeneous catalysis and in cost-effectiveness [38].

The reaction with ethyl propiolate in the presence of 0.9 mol % catalyst is very slow with a half conversion time of more than nine hours (Table 1, entry 4, yellow diamonds). We attribute this poor catalytic activity to the formation of copper acetylide clusters or even coordination polymers. Analogous dicopper complexes of more sterically demanding bis-1,2,4-triazolylidene ancillary ligands are quantitatively converted to octacopper hexaacetylide clusters under the same conditions [37]. To date, we have not been able to characterize thiazolylidene copper acetylides. Addition of acetic acid greatly increases the rate of the CuAAC reaction with ethyl propiolate, so that half-conversion is reached after 6 min (Table 1, entry 5, red squares). These observations are again consistent with the formation of a thermodynamically stable copper acetylide species [37], which are regenerated in the presence of acid to catalytically active dicopper acetylide intermediates [36]. The reaction catalyzed by copper acetate proceeds very slow. The extrapolated half-conversion is reached within approximately one day (Table 1, entry 6, grey triangles).

## Conclusion

In summary, we have presented a molecularly defined bisthiazolylidene dicopper(I) complex that features high catalytic activity in CuAAC reactions. Its three-step synthesis is straightforward and cost-efficient. The modular design of this class of catalysts renders possible the tuning of the complex’s properties and its features according to specific demands. Dicopper complexes with thiazolylidene ancillary ligands provide for improved availability, air-stability and convenience for the growing community of CuAAC users.

## Experimental

### General methodology

All reactions were carried out, unless described otherwise, under normal laboratory conditions under air. Reactions involving air-sensitive reagents were carried out in an atmosphere of argon using standard Schlenk techniques or in an MBraun LABmaster 130 glove box operated with nitrogen. Chemicals and solvents used in this work were supplied by the Department of Chemistry at the Ruprecht-Karls-University Heidelberg or bought directly from Acros Organics, Fisher Scientific, Sigma Aldrich, Strem Chemicals, and TCI. Anhydrous solvents were taken from an MBraun MB SCS-800 solvent purification system containing appropriate drying agents. Deuterated solvents for the use of NMR spectroscopy were obtained from Deutero GmbH and Euriso-Top.

^1^H NMR spectra were recorded at room temperature and the following instruments were employed: Bruker ARX-250 (250 MHz), Bruker Avance 300 (300 MHz), Bruker Avance 400 (400 MHz), Bruker Avance 500 (500 MHz). Chemical shifts δ are indicated in ppm and were determined by reference to the residual ^1^H solvent peaks (acetone: 2.05 ppm; chloroform: 7.26 ppm; dichloromethane: 5.32 ppm; DMSO: 2.50 ppm). Coupling constants *J* are given in Hz. The following abbreviations describe the observed multiplicities: s = singlet, d = doublet, t = triplet, q = quartet, quin = quintet, sext = sextet, sept = septet, m = multiplet (composed abbreviations refer to multiple coupling patterns with the first letter indicating the greater coupling constant). ^13^C{^1^H} NMR spectra were recorded at room temperature with the following spectrometers: Bruker Avance 300 (75 MHz) and Bruker Avance 500 (125 MHz). The spectra were calibrated with respect to the solvent (acetone: 29.84 ppm, 206.26 ppm; chloroform: 77.16 ppm; dichloromethane: 53.84 ppm; DMSO: 39.52 ppm). For processing, analysis and interpretation of NMR spectra, the program TopSpin 3.2 by Bruker was used. All observed signals are singlets. Elemental analyses were carried out in the Department of Chemistry at the University of Heidelberg on the instruments vario EL and vario MICRO cube by Elementar Analysensysteme GmbH. Infrared spectra were recorded on a Bruker Lumos instrument with a Germanium ATR-crystal. The positions of the peaks are indicated in wavenumbers ν in cm^–1^. The following abbreviations were used to describe both the intensity and profile of the signals: w (weak), m (medium), s (strong), br (broad). Mass spectra were recorded by the Mass Spectrometry Service Facility of the Organic-Chemical Department of the University of Heidelberg using the following instruments: Vacuum Generators ZAB-2F, Finnigan MAT TSQ 700, JEOL JMS-700, Bruker ICR Apex-Qe hybrid 9.4 T FT-ICR. In general the ionization method was specified. Apart from the method of ionization and the peak of the molecular ion, the base peak and characteristic fragmentation peaks with their relative intensities are reported.

### Syntheses

#### 3,3'-(Ethane-1,2-diyl)bis(4,5-dimethylthiazolium) dibromide (**1a**)

A Schlenk flask was charged with 3.00 equiv 4,5-dimethylthiazole (1.00 g, 8.84 mmol) and 1.00 equiv 1,2-dibromoethane (0.55 g, 2.95 mmol). The mixture was stirred at 115 °C for 3 d. After cooling to room temperature, the resulting solid was suspended in ethanol (2 mL) and the mixture was filtered. The solid residue was washed with ethanol (3 × 2 mL) and diethyl ether (3 × 3 mL) and dried in vacuo to give the beige product **1a** (1.05 g, 2.53 mmol, 86%). ^1^H NMR (400.33 MHz, DMSO-*d*_6_, 300.0 K) δ 9.90 (s, 2H, NC*H*S), 5.04 (s, 4H, C*H*_2_), 2.52 (s, 6H, NCC*H*_3_), 2.47 (s, 6H, SCC*H*_3_) ppm; ^13^C{^1^H} NMR (100.66 MHz, DMSO-*d*_6_, 295.0 K) δ 157.1 (N*C*HS), 141.9 (N*C*CH_3_), 133.7 (S*C*CH_3_), 50.6 (*C*H_2_), 12.1 (NC*C*H_3_), 11.1 (SC*C*H_3_) ppm; elemental analysis calculated: C, 34.80; H, 4.38; N, 6.76; found: C, 34.69; H, 4.63; N, 6.64; HRMS (ESI^+^, DMSO/MeOH) *m*/*z* (%): 253.0827 (100.0) [M – H − 2Br]^+^, 352.9222 (41.3), 746.9344 (35.0) [2 M(^79^Br) − Br]^+^, calculated for [M – H − 2Br]^+^: 253.0833, found: 253.0827; IR (ATR) ν = 3400 (w, br), 3070 (s), 2970 (s), 1738 (m), 1583 (s), 1443 (s), 1405 (s), 1189 (s), 798 (s) cm^−1^; mp 255 °C dec.

#### 3,3'-(Ethane-1,2-diyl)bis(4,5-dimethylthiazolium) bis(hexafluorophosphate) (**1b**)

1.00 equiv 3,3'-(ethane-1,2-diyl)bis(4,5-dimethylthiazolium) dibromide (**1a**, 1.50 g, 3.62 mmol) was dissolved in 75 mL H_2_O. The solution was added slowly to aqueous 55% hexafluorophosphoric acid (3.00 equiv, 2.88 g, 10.9 mmol) in 50 mL H_2_O. The mixture was stirred at room temperature for 3 h. The formed precipitate was filtered and washed with water (3 × 25 mL) and diethyl ether (5 × 20 mL). It was dried in vacuo to give the colourless product **1b** (1.50 g, 2.75 mmol, 76%). Single crystals of salt **1b** that were suitable for an X-ray structure analysis were obtained from acetone/diethyl ether. ^1^H NMR (400.33 MHz, acetone-*d*_6_, 295.0 K) δ 9.88 (s, 2H, NC*H*S), 5.42 (s, 4H, C*H*_2_), 2.68 (s, 6H, NCC*H*_3_), 2.66 (s, 6H, SCC*H*_3_) ppm; ^13^C{^1^H} NMR (100.66 MHz, acetone-*d*_6_, 295.0 K) δ 157.1 (N*C*HS), 143.7 (N*C*CH_3_), 136.1 (S*C*CH_3_), 52.4 (*C*H_2_), 12.6 (NC*C*H_3_), 11.6 (SC*C*H_3_) ppm; elemental analysis calculated: C, 26.48; H, 3.33; N, 5.15; found: C, 26.50; H, 3.34; N, 5.37; HRMS (ESI^+^, CH_2_Cl_2_/MeOH) *m*/*z* (%): 253.0828 (14.0) [M – H − 2PF_6_]^+^, 399.0548 (100.0) [M − PF_6_]^+^, 683.1201 (15.7), 943.0741 (73.0) [2 M − PF_6_]^+^; calculated for [M − PF_6_]^+^: 399.0548, found: 399.0548; IR (ATR) ν = 3132 (w), 1739 (w), 1595 (w), 1453 (w), 1211 (w), 828 (s), 740 (w) cm^−1^; mp 220 °C dec.

#### μ-Acetato-κ*O*,κ*O*'-μ-[3,3'-(ethane-1,2-diyl)bis(4,5-dimethylthiazol-2-ylidene)]-κ*C*,κ*C*'-dicopper(I) hexafluorophosphate (**2**)

A Schlenk flask flushed with argon was charged with 3,3'-(ethane-1,2-diyl)bis(4,5-dimethylthiazolium) bis(hexafluorophosphate) (**1b**, 0,10 g, 0.18 mmol, 1.00 equiv) and anhydrous sodium acetate (0.04 g, 0.42 mmol, 2.30 equiv). The reaction mixture was stirred under reduced pressure overnight. In a glove box, copper(I) acetate (0.05 g, 0.40 mmol, 2.20 equiv) and dichloromethane or tetrahydrofuran (3 mL) were added. The suspension was stirred at room temperature for 5 d.

a) Procedure for the reaction in dichloromethane: The suspension was filtered over a frit and the solution was concentrated by reducing the solvent in vacuo to 2 mL. Diethyl ether (4 mL) was added and the formed precipitate was filtered, washed with diethyl ether (3 × 2 mL) and dried in vacuo to give the light beige product **2** (0.06 g, 0.10 mmol, 56%).

b) Procedure for reaction in tetrahydrofuran: The solvent was removed under reduced pressure and dichloromethane (4 mL) was added in the glove box. The suspension was filtered over a frit and the solution was concentrated by reducing the solvent in vacuo to 2 mL. Diethyl ether (4 mL) was added and the formed precipitate was filtered, washed with diethyl ether (3 × 2 mL) and dried in vacuo to give the light beige product **2** (0.09 g, 0.16 mmol, 86%). ^1^H NMR (600.24 MHz, CD_2_Cl_2_, 295.0 K) δ 4.85 (s, 4H, C*H*_2_), 2.47 (s, 6H, NCC*H*_3_), 2.38 (s, 6H, SCC*H*_3_), 2.13 (s, 3H, *H*_3_CCOO) ppm; ^13^C{^1^H} NMR (150.95 MHz, CD_2_Cl_2_, 295.0 K) δ 197.7 (N*C*CuS), 182.7 (H_3_C*C*OO), 141.2 (N*C*CH_3_), 133.6 (S*C*CH_3_), 55.3 (*C*H_2_), 22.9 (H_3_*C*COO), 12.7 (NC*C*H_3_), 12.1 (SC*C*H_3_) ppm; elemental analysis calculated: C, 28.82; H, 3.28; N, 4.80; found: C, 28.70; H, 3.61; N, 4.84; HRMS (ESI^+^, DMSO/MeOH) *m*/*z* (%): 251.0672 (42.3) [M − 2Cu – H – Oac − PF_6_]^+^, 283.0935 (100.0) [M − 2Cu – Oac − PF_6_ + CH_3_O]^+^, 397.0392 (17.7), 599.0698 (69.9); calculated for [M − 2Cu – Oac − PF_6_ + CH_3_O]^+^: 283.0949, found: 283.0935. IR (ATR) ν = 1594 (w), 1555 (m), 1447 (m), 1397 (w), 1328 (w), 835 (s), 691 (m) cm^−1^; mp 175 °C dec.

## Supporting Information

File 1 Author contributions, details of the procedures for the kinetic measurements, and figures of NMR spectra.
