# Supplementary material for: Dinuclear thiazolylidene copper complex as highly active catalyst for azid–alkyne cycloadditions
Source: Beilstein J Org Chem. 2016 Jul 21;12:1566–72. doi: 10.3762/bjoc.12.151 (PMC4979731; doi:10.3762/bjoc.12.151)
Supplement: File 1 — Author contributions, details of the procedures for the kinetic measurements, and figures of NMR spectra. [file Beilstein_J_Org_Chem-12-1566-s001.pdf]

Supporting Information

for

Dinuclear thiazolylidene copper complex as highly active catalyst for azid–alkyne cycloadditions

Anne L. Schöffler, Ata Makarem, Frank Rominger and Bernd F. Straub*

Address: Organisch-Chemisches Institut, Universität Heidelberg, Im Neuenheimer Feld 270, D-69120 Heidelberg, Germany

Email: straub@oci.uni-heidelberg.de

*Corresponding author

In memoriam Prof. Dr. Peter Hofmann

Author contributions, details of the procedures for the kinetic measurements, and figures of NMR spectra

Author contributions

M. Sc. Anne L. Schöffler synthesized the new compounds, analyzed their spectroscopic data and wrote the major part of the manuscript and of the Supporting Information. Dr. Ata Makarem contributed some advice concerning the synthesis and purification of the reported copper complexes and passed on the procedural knowledge for kinetic measurements. Dr. Frank Rominger analyzed the single crystal X-ray structure. Prof. Dr. Bernd F. Straub contributed the idea of using thiazoles as azole starting compound, mentored the experimental work, wrote parts of the manuscript, and contributed to some figures.

Kinetic measurements

General information

The kinetics of CuAAC reactions of benzyl azide with phenylacetylene or ethyl propiolate was studied by ^{1}H NMR spectroscopy.. Due to the highly exothermic nature of the triazole formation, a low substrate concentration is essential to ensure an isothermal reaction and to prevent a thermal runaway. In a blind test, we observed that acetic acid alone in the absence of a copper complex does not catalyze the CuAAC reaction. Degassed deuterated dichloromethane (stored over molecular sieves 4 Å) was used as solvent. The reaction mixtures were prepared in an MBraun LABmaster 130 glove box under an atmosphere of nitrogen using J. Young NMR tubes and microliter syringes. Stock solutions were prepared when direct additions of determined amounts of reagents or chemicals into the NMR tube were inaccurate. The sample tubes were brought to the NMR spectrometer immediately after the reaction started in the glove box. The time differences between the start of the reaction and the recording of a spectrum were measured by watch. All of the weighed amounts are accurate within an error of ±0.1 mg that refers to the scale instrument.

Click reaction of benzyl azide and phenylacetylene

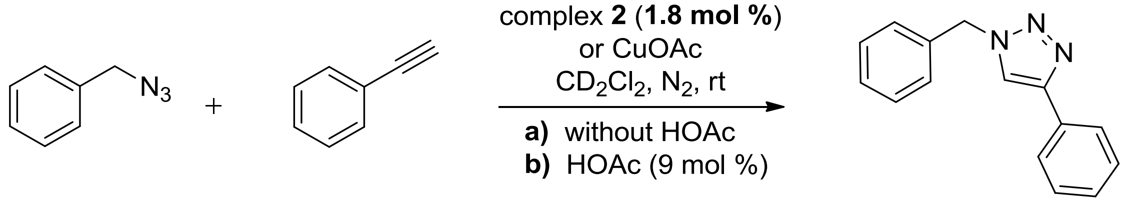

Sample preparation

Stock solution **A**

1.0 mg (1.78 µmol) complex **2** was dissolved in 0.10 mL CD_2Cl_2.

Stock solution **B**

2.5 µL (44.4 µmol) acetic acid was diluted in 0.10 mL CD_2Cl_2.

Saturated solution of **copper(I) acetate**

10.0 mg (81.6 µmol) copper(I) acetate was suspended in 1.0 mL CD_2Cl_2 and mixed properly. After 30 min the insoluble amount of copper(I) acetate had precipitated and the homogeneous saturated CuOAc solution was ready to use.

A J. Young NMR tube was charged with phenylacetylene (5.4 µL, 49.3 µmol), benzyl azide (6.4 µL, 49.3 µmol) and **a)** 0.45 mL CD_2Cl_2 for reaction **without acetic acid** or **b)** 0.44 mL CD_2Cl_2 and 10 µL stock solution **B** (HOAc in CD_2Cl_2, contains 9 mol % with respect to the substrates) for reaction **with acetic acid**. A ^{1}H NMR spectrum was recorded from the sample tube in order to check the purity. Then, by adding 50 µL of fresh stock solution **A** (complex **2** in CD_2Cl_2, contains 1.8 mol % with respect to the substrates) or 50 µL of fresh saturated solution of **copper(I) acetate** to the reaction mixture, the reaction was started.

NMR technique

The kinetics were measured by a continuous 1D-^{1}H NMR method, which is based on separated spectra. For the kinetic tests on a Bruker Avance spectrometer (300.51 MHz or 500.13 MHz) at 25 °C, a sequence of 1D-^{1}H NMR spectra with pulse 30° (flip angle) [1] and a fixed interval time between each two spectra was recorded. In each spectra sequence, the time difference between reaction start time in glove box and end of the first spectrum recording was determined by watch.

In the time-conversion diagrams each time-point shows the end of one NMR recording. For each ^{1}H NMR spectrum, the reaction conversion was calculated individually by using the intensities of the methylene groups of the benzyl compound (reactant: δ = 5.57 ppm, product: δ = 4.35 ppm).

Click reaction of benzyl azide and ethyl propiolate

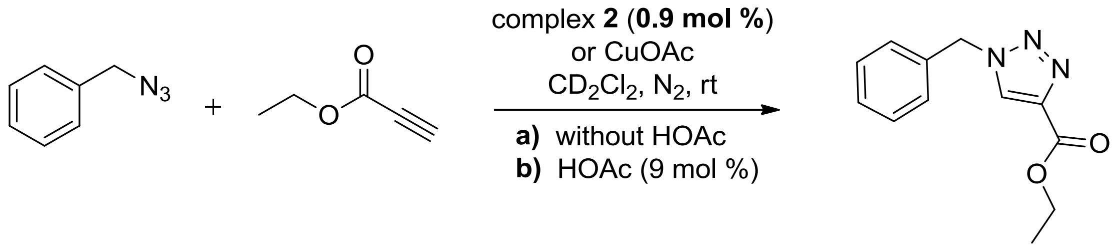

Sample preparation

Stock solution **A**

1.0 mg (1.78 µmol) complex **2** was dissolved in 0.20 mL CD_2Cl_2.

Stock solution **B**

2.5 µL (44.4 µmol) acetic acid was diluted in 0.10 mL CD_2Cl_2.

Saturated solution of **copper(I) acetate**

10.0 mg (81.6 µmol) copper(I) acetate was suspended in 1.0 mL CD_2Cl_2 and mixed properly. After 30 min the insoluble amount of copper(I) acetate had precipitated and the homogeneous saturated CuOAc solution was ready to use.

A J. Young NMR tube was charged with ethyl propiolate (5.0 µL, 49.3 µmol), benzyl azide (6.4 µL, 49.3 µmol) and **a)** 0.45 mL CD_2Cl_2 for reaction **without acetic acid** or **b)** 0.44 mL CD_2Cl_2 and 10 µL stock solution **B** (HOAc in CD_2Cl_2, contains 9 mol % with respect to the substrates) for reaction **with acetic acid**. A ^{1}H NMR spectrum was recorded from the sample tube in order to check the purity. Then, by adding 50

μL of fresh stock solution **A** (complex **2** in CD_2Cl_2, contains 0.9 mol % with respect to the substrates) or 50 μL of fresh saturated solution of **copper(I) acetate** to the reaction mixture, the reaction was started.

NMR technique

The kinetics were measured on a Bruker Avance spectrometer (300.13 MHz) at 25 °C using continuous NMR spectroscopy. Measurements have been recorded as pseudo-2D-spectra, where the chemical shift is displayed on the abscissa and the time dimension on the ordinate. The relaxation delay time D_1 in the pulse sequence has been chosen to be 115 seconds [2]. This comparatively long relaxation time has been chosen in order to allow for complete longitudinal relaxation of all protons before the next 90° pulse and thus ensure the measured intensities to be comparable to each other. The peaks whose intensities are used for calculating the conversion are the signals of the alkyne or alkene proton of the ester compound (reactant: δ = 2.95 ppm, product: δ = 8.00 ppm).

In the time-conversion diagrams, the time between each two time-points is the sum of the interval time and the NMR recording duration. The time difference between the start of a reaction in the glove box and the end of the first spectrum recording was determined by watch.

1. Makarem, A.; Berg, R.; Rominger, F.; Straub, B. F. *Angew. Chem. Int. Ed.* **2015,** *54*, 7431-7435. doi:10.1002/anie.201502368
2. Makarem, A. Complex Chemistry of Dicopper Click Catalysts. Ph.D. Thesis, Ruprecht-Karls-University Heidelberg, Germany, 2015.

NMR spectra

3,3'-(Ethane-1,2-diyl)bis(4,5-dimethylthiazolium) dibromide (1a)

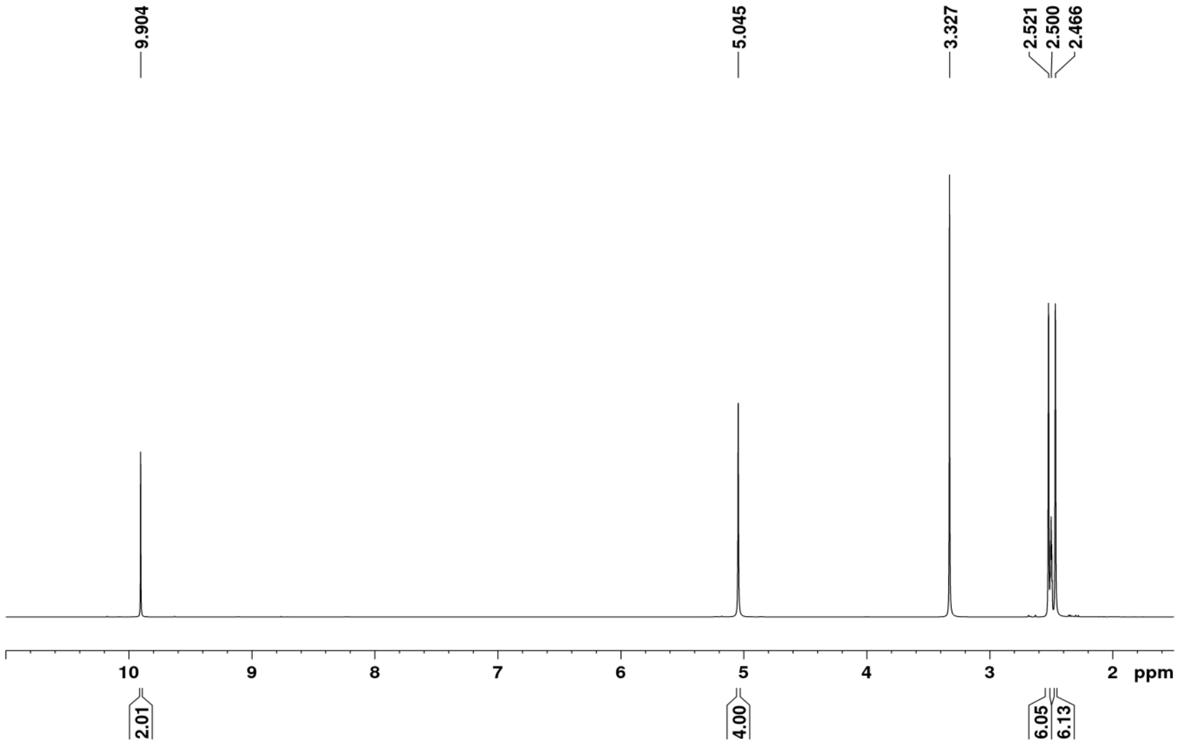

Figure 1: ^{1}H NMR spectrum of salt **1a** (400.33 MHz, DMSO-d_6, 300.0 K).

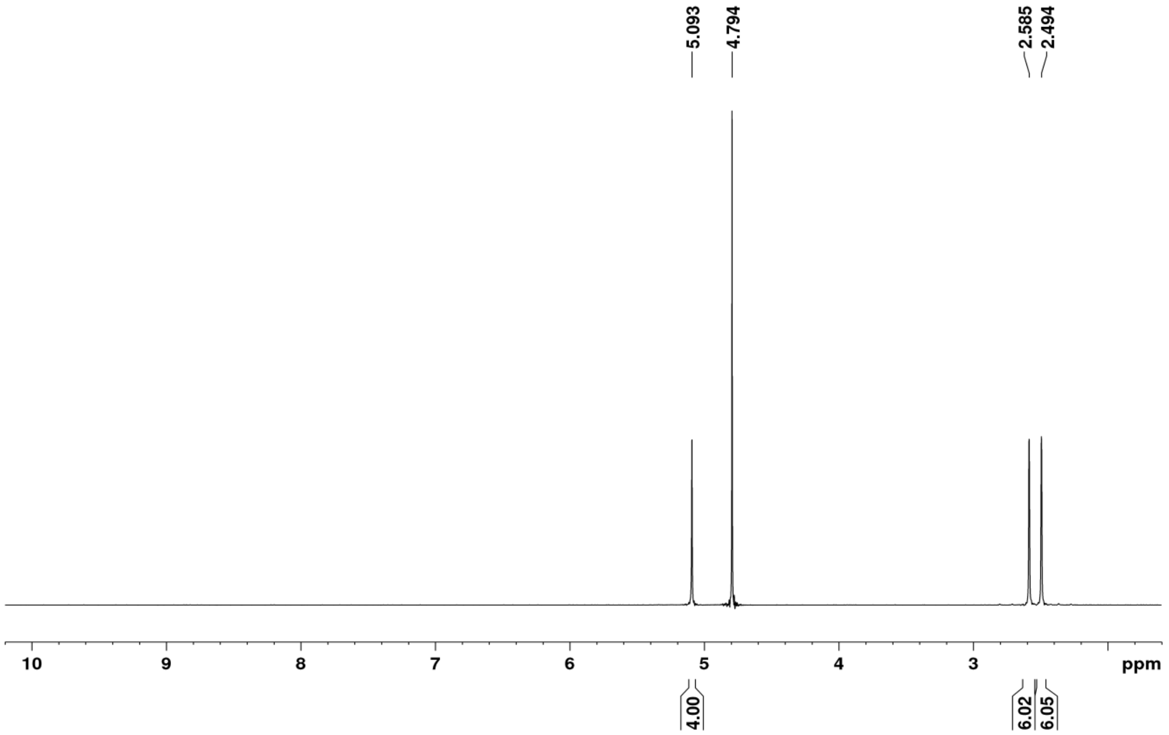

Figure 2: ^{1}H NMR spectrum of salt **1a** (300.51 MHz, D_2O, 300.0 K).

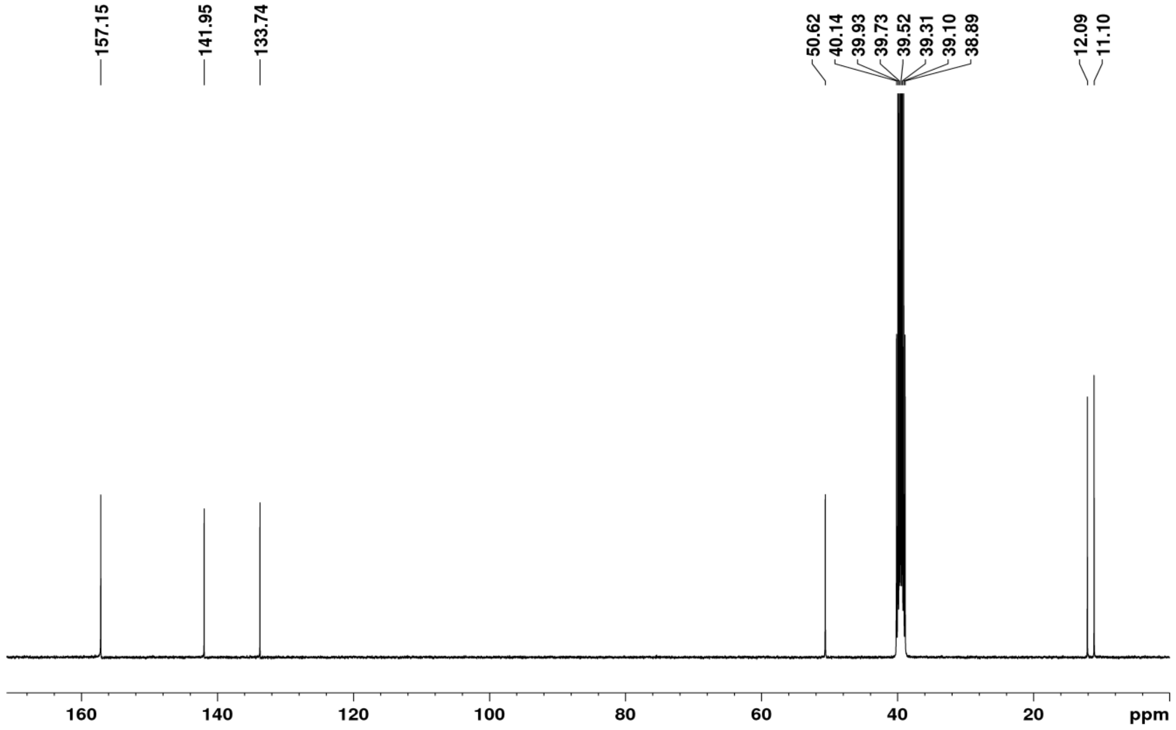

Figure 3: ^{13}C{^{1}H} NMR spectrum of salt **1a** (100.66 MHz, DMSO-d_6, 295.0 K).

3,3'-(Ethane-1,2-diyl)bis(4,5-dimethylthiazolium) bis(hexafluorophosphate) (1b)

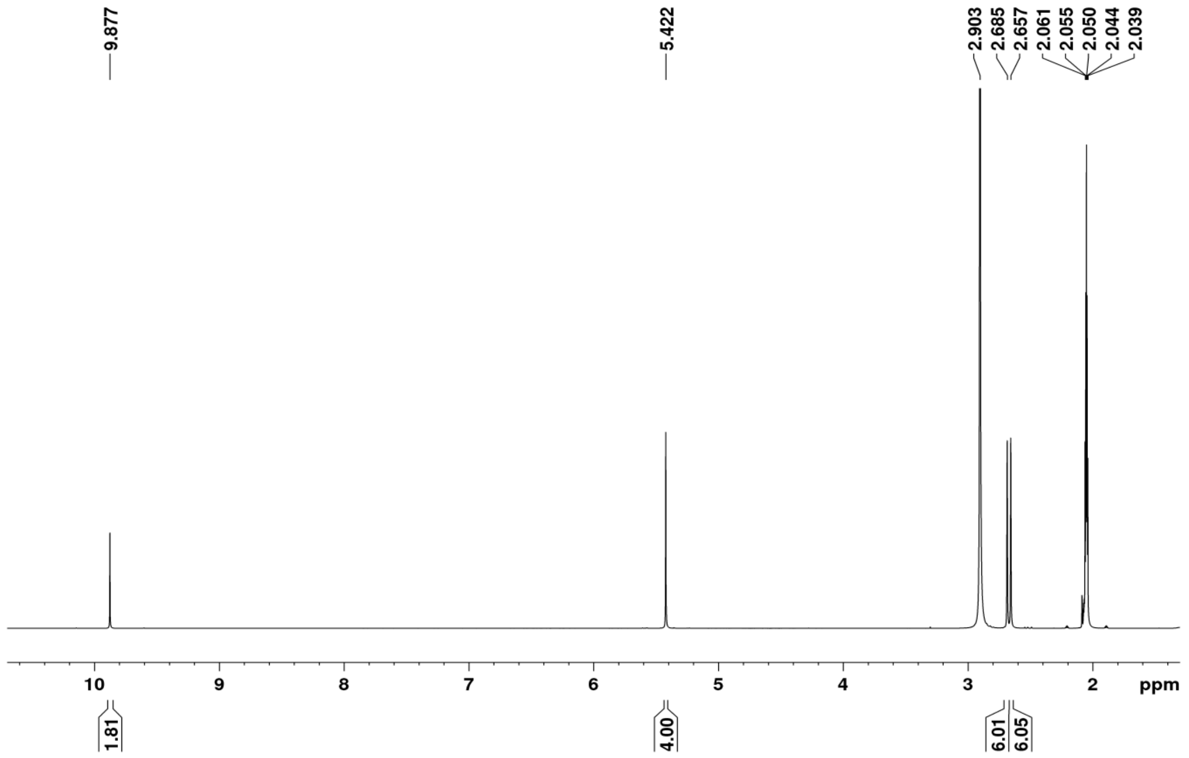

Figure 4: ^{1}H NMR spectrum of salt **1b** (400.33 MHz, acetone-d_6, 295.0 K).

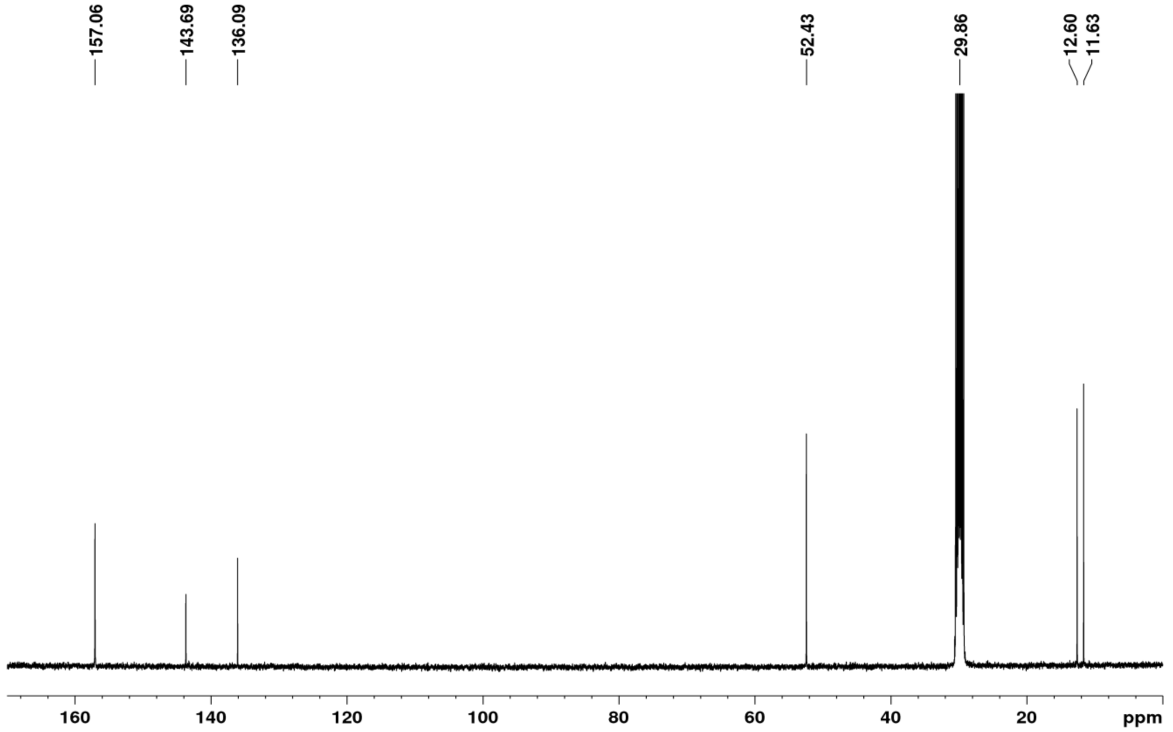

Figure 5: $^{13}C\{^{1}H\}$ NMR spectrum of salt **1b** (100.66 MHz, acetone-d_6, 295.0 K).

μ-Acetato-κ*O*,κ*O*'-μ-[3,3'-(ethane-1,2-diyl)bis(4,5-dimethylthiazol-2-ylidene)]-κ*C*,κ*C*'-dicopper(I) hexafluorophosphate (2)

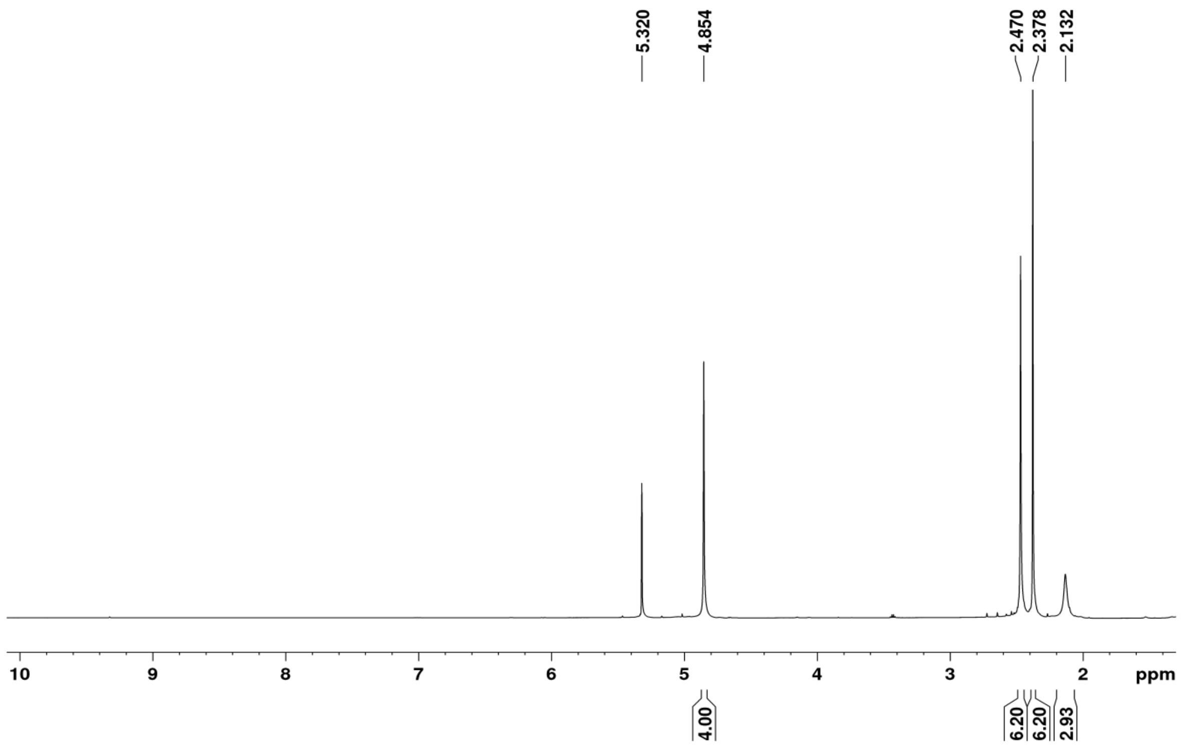

Figure 6: ^{1}H NMR spectrum of dicopper complex **2** (600.24 MHz, CD_2Cl_2, 295.0 K).

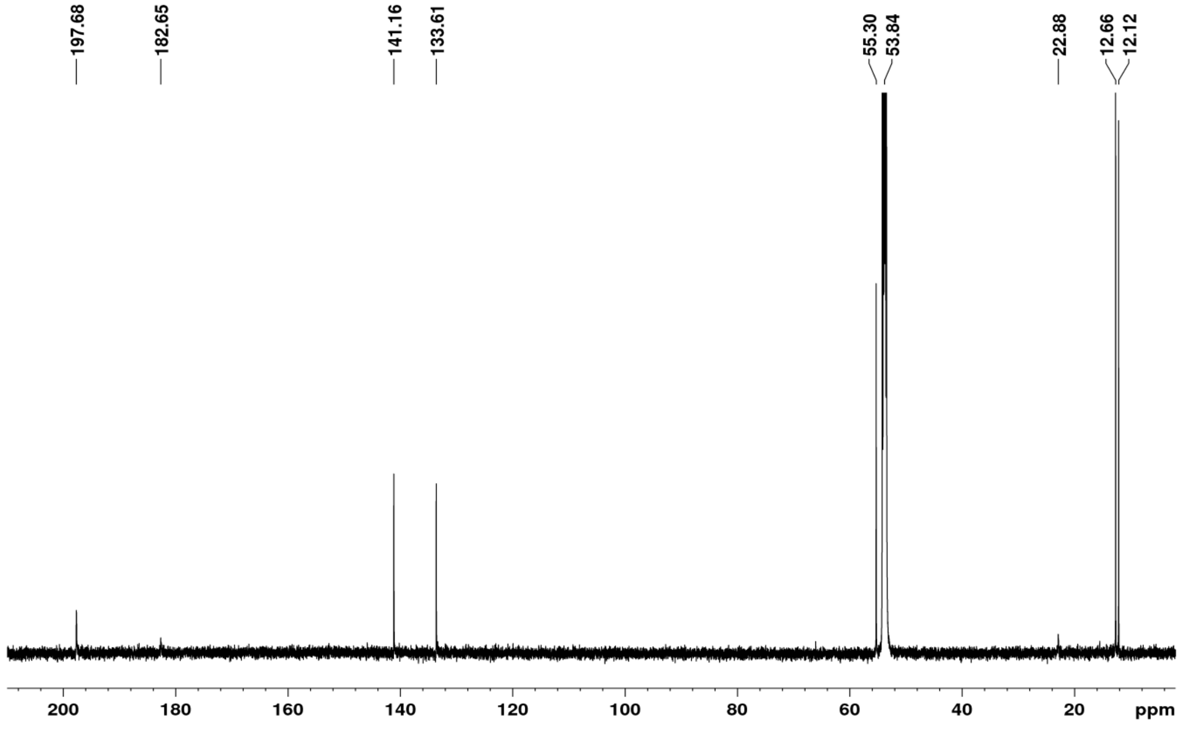

Figure 7: $^{13}C\{^1H\}$ NMR spectrum of dicopper complex **2** (150.95 MHz, CD_2Cl_2, 295.0 K).

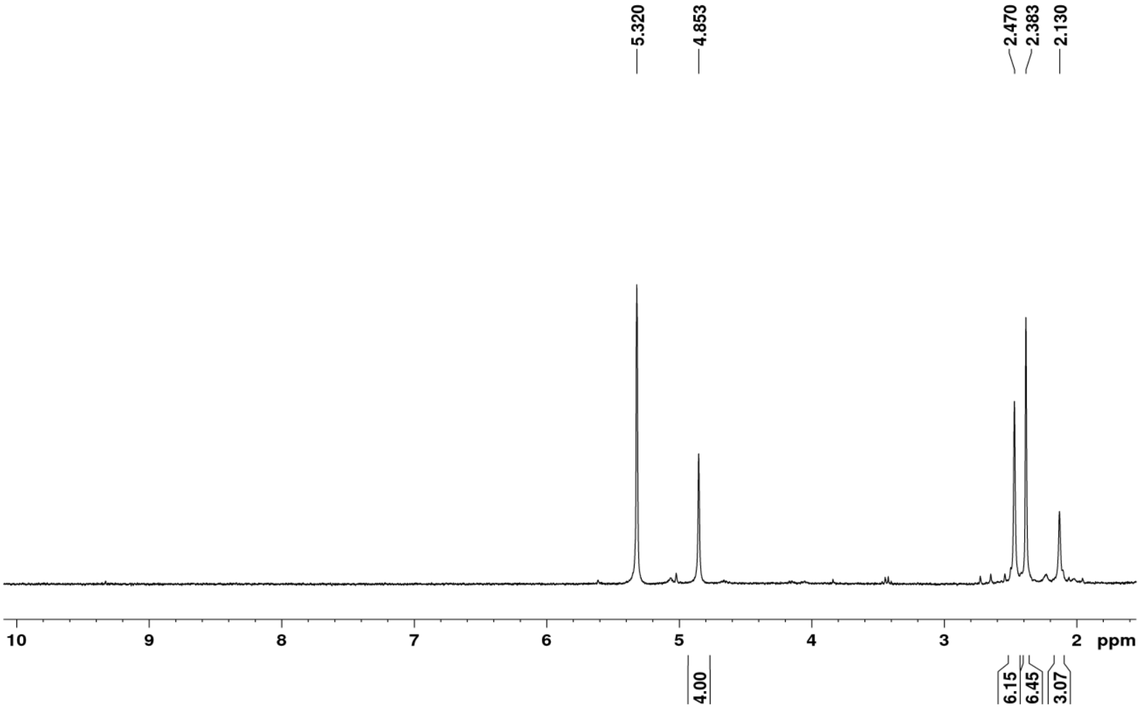

Figure 8: ^{1}H NMR spectrum of dicopper complex **2** after 6 d under an atmosphere of air (300.51 MHz, CD_2Cl_2, 300.0 K).
